# Global pattern and evolution of PPARγ in breast cancer: a 25-Year bibliometric and visualized knowledge map analysis

**DOI:** 10.3389/fonc.2026.1908399

**Published:** 2026-08-24

**Authors:** Xiaotian Zhang, Juan Jin, Yuan Cai, Xiaohui Luo, Qing She

**Affiliations:** 1 Department of Pathology, Baoji Central Hospital, Baoji, China; 2 Department of Urology, Baoji Central Hospital, Baoji, China; 3 Department of Breast, Baoji Central Hospital, Baoji, China

**Keywords:** bibliometric, breast cancer, PPARγ, randomized controlled trials, visualized

## Abstract

Bibliometric methods were employed to visually examine the global trends and progression of PPARγ in the breast with the aim of clarifying the evolution of significant academic contributions, collaborative networks, foundational disciplines, and key terms. A comprehensive search was conducted on publications related to PPARγ in breast cancer research using the Web of Science Core Collection database (WOSCC) and PubMed. The reference types for the WOSCC database were restricted to articles and reviews published in English, whereas PubMed focused on randomized controlled trials (RCTs). Data visualization and analysis were conducted using bibliometric tools, including CiteSpace, VOSviewer, Bibliometrix, and Scimago Graphica. Sensitivity analysis for non-deterministic indicators and bias risk assessment of RCTs were also conducted. A total of 1,391 publications and 3 RCTs were included in this study. The annual growth rate of publications in this field was 3.54%. The primary contributing countries were the United States, China, Japan, and Italy. The top three authors with the highest academic contributions were Catalano, Stefania, Bonofiglio, Daniela, and Ando, Sebastiano, which were also the most frequently cited authors. The most frequent keywords were PPARγ, breast cancer, apoptosis, and expression. This study provides the first bibliometric overview of PPARγ research in breast cancer, highlighting a continuous increase in scholarly output, with major contributions from Europe, the Americas, and Asia. The discipline has gradually evolved from initial basic and preclinical studies to exploring the complex regulatory system of the “cancer - metabolism - immunity” interaction network. Currently, the effectiveness of conventional PPARγ agonists in clinical trials is still limited, highlighting the urgent need and potential for the clinical development of highly selective PPARγ modulators. This review aims to deepen our understanding of the role of PPARγ in breast cancer, support the translation of related research into clinical practice, and offer a theoretical basis and directional guidance for future advancements in this field.

## 1 Introduction

According to global cancer statistics for 2022, breast cancer is the second most common cancer worldwide, ranking fourth in terms of mortality, and is the primary cause of cancer-related deaths among women (1). Factors such as *BRCA* mutations, old age, obesity, and endocrine levels can trigger the development of breast cancer (2, 3). This type of cancer is genetically diverse and can be divided into four subtypes: Luminal type A, Luminal type B, HER2 overexpression type, and triple-negative subtype (TNBC). Each subtype has distinct molecular pathological features and requires specific treatment approaches (4). TNBC is known for its resistance to endocrine and targeted therapies, as well as its increased invasiveness and tendency to metastasize (5). Breast cancer cells rely on the reprogramming of lipid metabolism to effectively adjust metabolic pathways during disease progression, meeting the energy needs for proliferation, invasion, metastasis, and responses to signaling molecules in the tumor microenvironment (6). Therefore, targeting the lipid metabolism in breast cancer may be a promising therapeutic approach.

In current clinical practice, breast cancer treatment has evolved into an era of “multidisciplinary, comprehensive treatment.” Treatment plans are highly personalized, based on molecular typing, the clinical stage of breast cancer, and the patient’s overall condition. As a result, a combination of radiotherapy, chemotherapy, endocrine therapy, targeted therapy, and surgical intervention is use (7–9). For HER2 positive and TNBC, preoperative systemic treatment has become standard to achieve the dual goals of “downstaging and lactation preservation” and “drug sensitivity touchstone” (10, 11). Sentinel lymph node biopsy (SLNB) is the “gold standard” minimally invasive technique for assessing metastasis in axillary lymph nodes (12). Recent clinical trials have shown that for patients with 1–2 positive lymph nodes identified in SLNB, omitting axillary lymph node dissection (ALND) is safe. Additionally, for patients with 3–5 positive lymph nodes, ALND does not affect overall survival (OS) (13). Surgical methods primarily include breast-conserving surgery and mastectomy. Traditional radical surgery (Halsted surgery) used in the past is highly invasive and is rarely performed (14). Modified radical mastectomy is the most commonly used mastectomy technique. This procedure involves removing all breast tissue, along with the fascia of the pectoralis major muscle and axillary lymph node dissection. Importantly, the pectoralis major and minor muscles are preserved, which enhances aesthetic outcomes and functional capacity (15). For patients needing postoperative breast reconstruction, total mastectomy with skin preservation or preservation of the nipple and areola can be performed alongside prosthetic reconstruction to optimize cosmetic results (16).

PPAR is a member of the nuclear receptor superfamily. It is activated upon ligand binding to G protein-coupled receptors, which subsequently stimulate downstream signal transducers, including adenylate cyclase and cyclic adenylate (17). This activation process facilitates the translocation of the nuclear receptor protein, PPAR, thereby modulating the expression of target genes. PPARγ, the most extensively studied member of the PPAR family, primarily regulates processes such as fatty acid metabolism and influences the progression of various metabolic disorders (18). Recent research suggests that PPARγ plays a significant role in the regulation of lipid metabolism in breast cancer cells. It concurrently induces the differentiation of cancer-associated fibroblasts (CAF) and adipose tissue, thereby affecting the remodeling of the tumor microenvironment (TME) and promoting energy metabolism to support tumor cell proliferation (19). PPARγ agonists are predominantly used for the hypoglycemic management of type 2 diabetes (20). Agonists such as pioglitazone and rosiglitazone have demonstrated cancer-suppressing effects by modulating the malignant biological behavior of breast cancer cells, including their proliferation and migration (21, 22). The PPARγ inhibitor GW9662 counteracts the anti-tumor effect by inhibiting autophagosome formation, impeding the apoptosis of tumor cells, enhancing T cell activity, and augmenting anti-tumor immunotherapy (23, 24).

Currently, there is a substantial body of evidence regarding the role of PPARγ in breast cancer. However, the complexity of an action network often leads to controversial conclusions. There is a notable lack of systematic reviews that visually integrate and analyze their evolutionary context, knowledge structure, and research frontiers. Consequently, this study seeks to elucidate development trends and identify core hotspots by systematically reviewing and quantitatively analyzing basic and clinical research in this field over the past few decades.

This work holds considerable scientific significance, as it seeks to resolve domain disputes, consolidate fragmented understandings, and provide empirical evidence and strategic guidance for pivotal future research directions.

## 2 Methods

### 2.1 Research methodologies

The WOSCC database mainly contains English documents, covering major academic research achievements worldwide, and has the best compatibility with major bibliometric tools (66). The PubMed database was used to identify RCTs that improved the clinical background. Data are obtained from WOSCC and the PubMed database, and retrieved using Boolean operators (“AND” and “or”). The search formula is as follows: (TS=“Breast Cancer” OR “Breast Neoplasm*” OR “Breast Tumor” OR “Breast Tumour” OR “Malignant Neoplasm of Breast”) AND (TS=“PPAR gamma” OR “PPARγ” OR “PPARG” OR “NR1C3” OR “Peroxisome Proliferator-Activated Receptor Gamma” OR “Peroxisome Proliferator-Activated Receptor γ”).

Inclusion criteria: 1. The time range of literature search is up to December 31, 2025; 2. The literature types was limited to articles and reviews. 3. The language requirement was English.

Exclusion criteria: 1. Republished literature. 2. Irrelevant document types, including but not limited to conference papers, editorials, letters, conference abstracts, book reviews, and corrections. 3. Preprint literature. 4. Withdrawn literature.

### 2.2 Data acquisition

**Figure 1:**
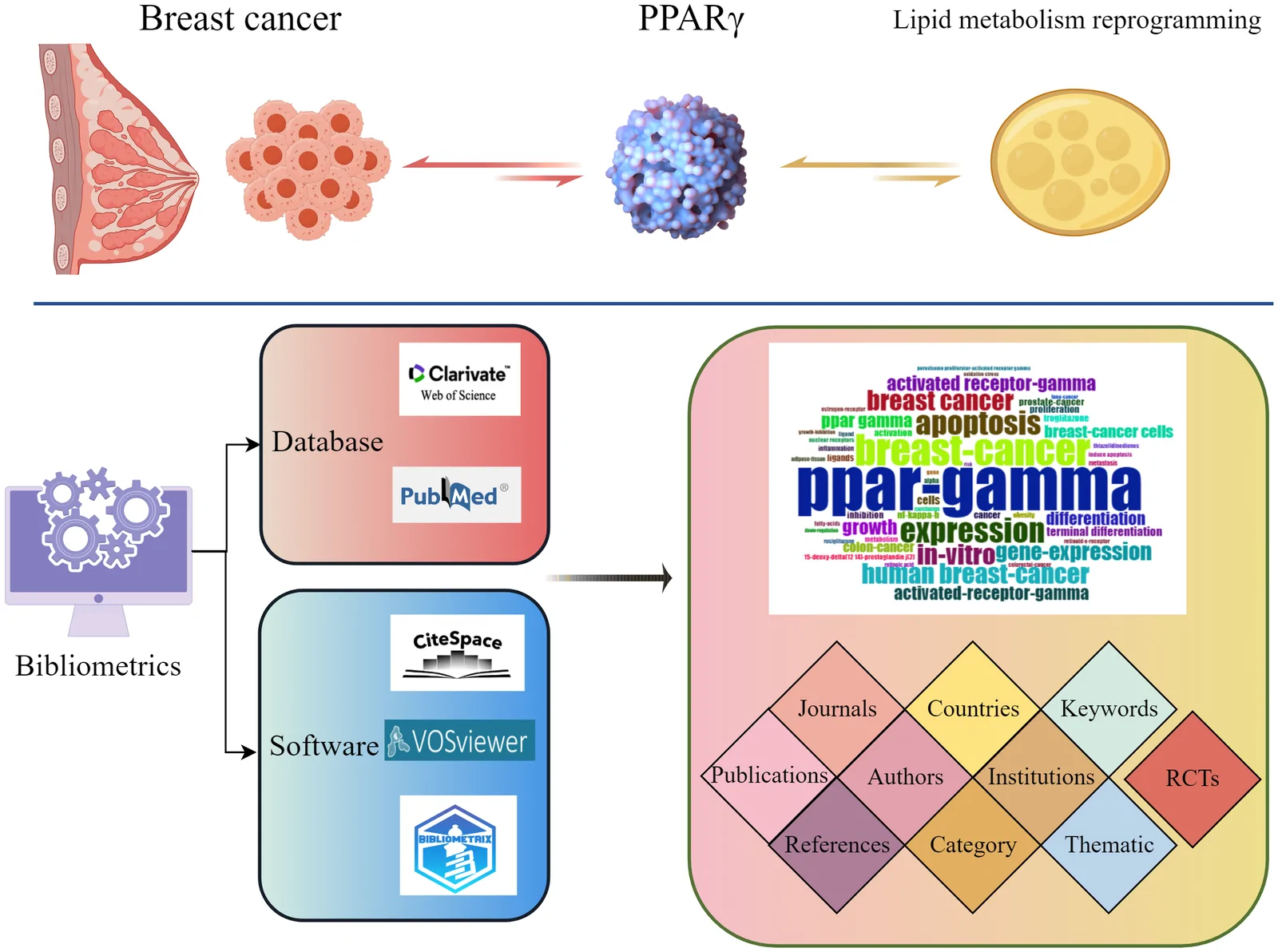
Flowchart of research methods and application tools.

**Figure 2:**
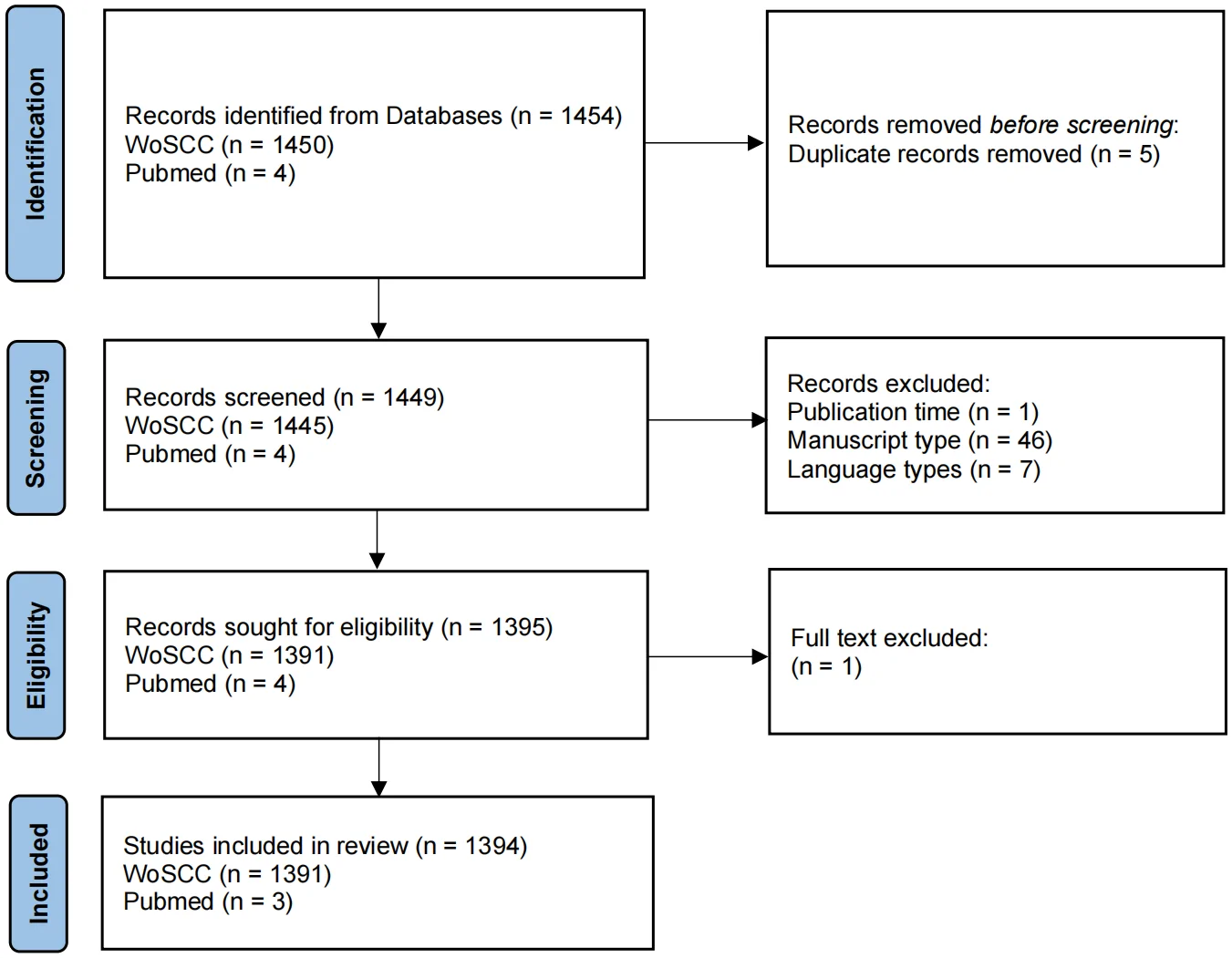
Flowchart of the literature search and study selection process.

Data were collected on January 22, 2026. A total of 1,450 documents were retrieved from the WOSCC database. Based on the established inclusion and exclusion criteria, one document failed to meet the year requirements, 46 did not satisfy the document type requirements, and seven did not meet the language requirements. Consequently, 1,396 documents were included in the study. These 1,396 documents were imported into CiteSpace, five of which were de-duplicated and subsequently excluded based on the years identified by CiteSpace. The remaining 1,391 documents were included in the study. Four RCTs were retrieved from the PubMed database. Two independent researchers assessed the risk of bias for each RCT using the Cochrane RoB 2 tool, following the PRISMA 2020 guidelines. Initial discrepancies were addressed through discussion, while any unresolved conflicts were adjudicated by a third investigator, who assigned the final risk of bias grade. Onlinemeta tool (https://smuonco.shinyapps.io/Onlinemeta/#shiny-tab-Home) was used to evaluate the Cochrane Risk of Bias. Among them, the horizontal axis represents the percentage of different documents, the vertical axis represents different evaluation items, green represents low risk, red represents doubtful risk and gray represents high risk. A flowchart of the literature search and study selection process is shown in **Figures 1**, **2**.

### 2.3 Software parameter

The complete record and citation data of the selected literature were exported from the WOSCC in the txt format (25). Bibliometrix 5.4.0 is a robust and comprehensive open-source software package in R specifically designed for bibliometric analyses. It facilitates basic statistical assessments and analyses of countries’ collaboration through a World Map. CiteSpace 7.0 performs citation burst detection, time series analysis, and journal dual-graph analysis, while VOSviewer 1.6.20 specializes in network visualization and cluster analysis. Developed by Dr. Chen, CiteSpace serves as a visualization analysis tool that reveals macroscopic dynamic patterns and evolutionary trends in scientific literature. The specific parameter settings for CiteSpace were as followed: Node types included cited journal, cited reference, category, and keyword. The selection criteria retain the top 50 items. Pruning algorithms, specifically “Pathfinder,” “Pruned slice networks,” and “Pruned the merge network,” were employed to simplify the network structure and highlight core connections. Parameters were set: time span (2000–2025), year per slice (1), and the link (strength: cosine, range: slice) and the selection criterion (g-index: k = 25). VOSviewer is a professional software tool used to construct and Visual bibliometric networks. The specific parameter settings for VOSviewer were as followed: Node types included author, cited author, institution, country, and keyword. The normalization method employed association strength. The layout parameters were as followed: Attraction = 2, Repulsion = 1 Clustering: Resolution = 1, Minimum cluster size = 1, Orientation: Rotation = 90 degrees. In the generated visualization model, the size of the nodes represents the frequency or reference count of each node, whereas the thickness of the connection lines between the nodes indicates the strength of the relationships between the items. Scimago Graphica Beta 1.0.53 is an open-source data visualization and scientific charting tool that has rapidly gained popularity in bibliometrics and scientific research data analysis.

### 2.4 Sensitivity analysis

This study employs an overall counting method to conduct a statistical analysis of deterministic indicators. The indicators extracted using Bibliometrix, including the number of publications, national publications, institutional publications, and author publications. et, exhibit strong stability and do not fluctuate with parameter disturbances. Consequently, they were unsuitable for sensitivity analysis. In contrast, non-deterministic indicators, such as reference bursts, keyword bursts were significantly influenced by parameter perturbation. To assess the robustness of the bibliometric structure, a sensitivity analysis was performed through parameter perturbation. By increasing the k-value in CiteSpace to determine the stability of the selected core nodes. The professional online data analysis and visualization platform Wei Sheng Xin (https://www.bioinformatics.com.cn/) was used to generate the Venn diagram and calculate the repetition rate of principal analysis and parameter disturbance. A repetition rate exceeding 80% was deemed very stable; 65% to 79% was considered acceptable; between 50% and 64% were regarded as critical, and below 50% was classified as unstable.

## 3 Results

### 3.1 Main information and annual quantitative trend of publications

**Figure 3:**
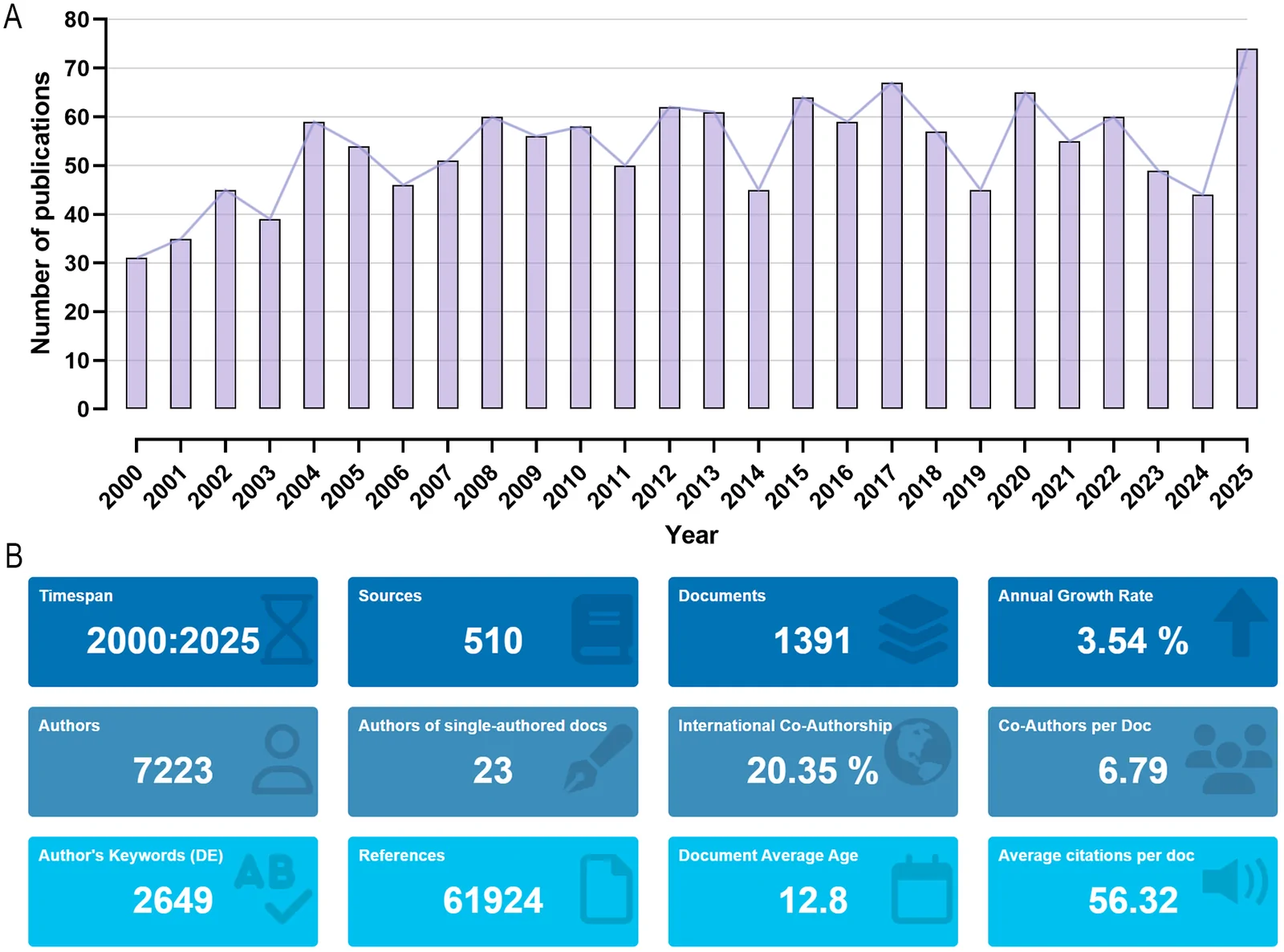
Annual quantitative trend of publications and main information. **(A)** Annual quantitative trend of publications. **(B)** Main information.

The annual publication volume of PPARγ in breast cancer exhibits fluctuations within a defined range, culminating in a significant increase in 2025, which marks a historical peak (**Figure 3A**). This research domain began in 2000 and produced 1,391 documents, reflecting an annual growth rate of 3.54%, thereby indicating a consistent upward trend in research activity. The field encompasses 7,223 authors, with an average of 6.79 authors per document. The level of international collaboration is notably high, with an international cooperation rate of 20.35%. Cumulatively, there were 61,924 citations, with an average of 56.32 citations per document (**Figure 3B**).

### 3.2 Countries and institutions

**Figure 4:**
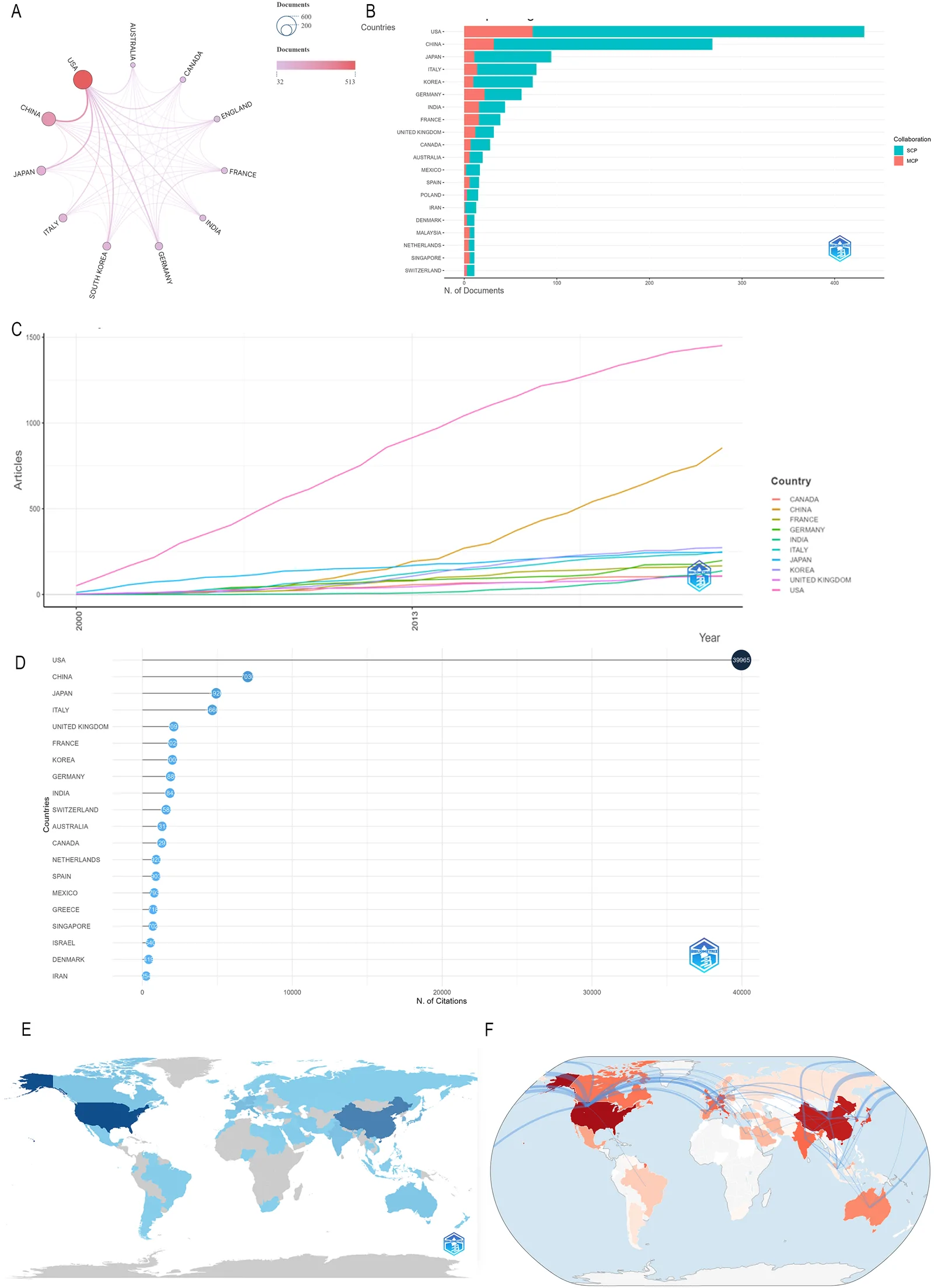
National publication volume, trend, and cooperative knowledge map. **(A)**The number of national publications. **(B)** Corresponding Author’s Countries. **(C)** Country Production over Time. **(D)** Most Cited Countries. **(E)** Country Scientific Production. The bluer the color of the map plate, the more posts it represents. **(F)** Countries’ Collaboration World Map. The redder the map plate is, the more posts it represents. The connecting line represents the cooperation between different countries, and its thick line represents the cooperation intensity.

A total of 73 countries and regions participated in this study. The five countries with the highest number of published papers are, in order, the United States, China, Japan, Italy, and South Korea, which align perfectly with the sources of the corresponding authors (**Figures 4A**, **B**). The temporal curve of national output indicates that the United States maintains a leading position, with a significant increase in publications which is consistent with its status as the country with the highest publication count. In contrast, China experienced a notable increase in publications since 2013 (**Figure 4C**). The top five countries with the highest citations were the United States, China, Japan, Italy, and the United Kingdom (**Figure 4D**). The world maps depicting the number of published documents and collaboration among various countries are presented in **Figures 4E**, **F**.

**Figure 5:**
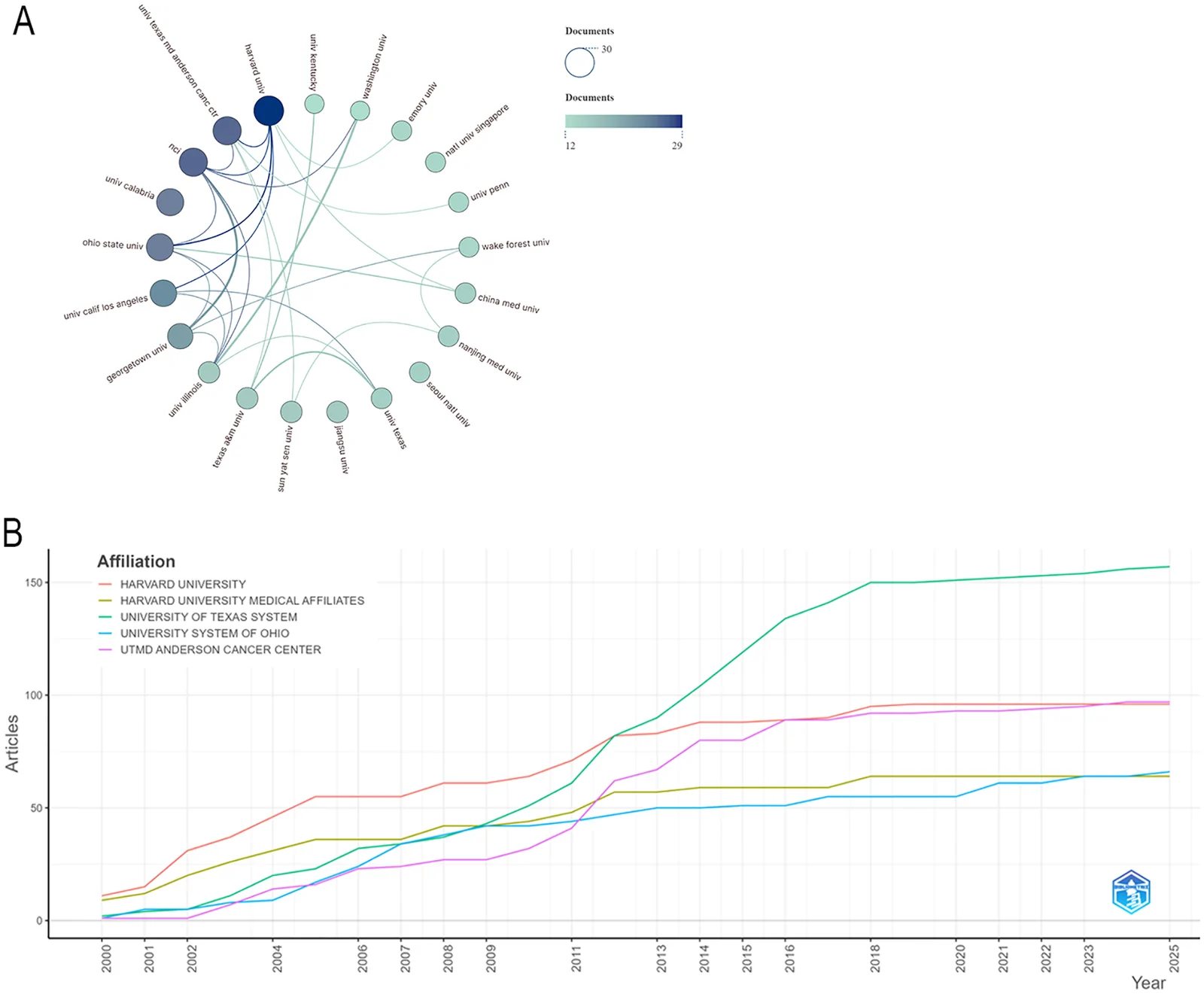
Institutions**’** publication volume and trend. **(A)** The number of institutional publications. **(B)** Institutions’ production over time. The size of the node and the blue color depth are directly proportional to the number of published articles. The connection lines represent the cooperation among different institutions.

In total, 490 institutions contributed to research in this field. The five institutions with the highest number of published papers were Harvard University, the University of Texas MD Anderson Cancer Center, the National Cancer Institute, the University of Calabria, and Ohio State University (**Figure 5A**). The curve graph depicting institutional output over time indicates that the cumulative publications from Harvard University and its affiliated medical schools solidify their status as leading institutions in terms of total publications, although their annual growth rate remains sluggish. In contrast, the University of Texas emerged as the institution with the highest number of publications after 2013; however, its growth rate has decelerated since 2018 (**Figure 5B**).

### 3.3 Authors and journals

**Figure 6:**
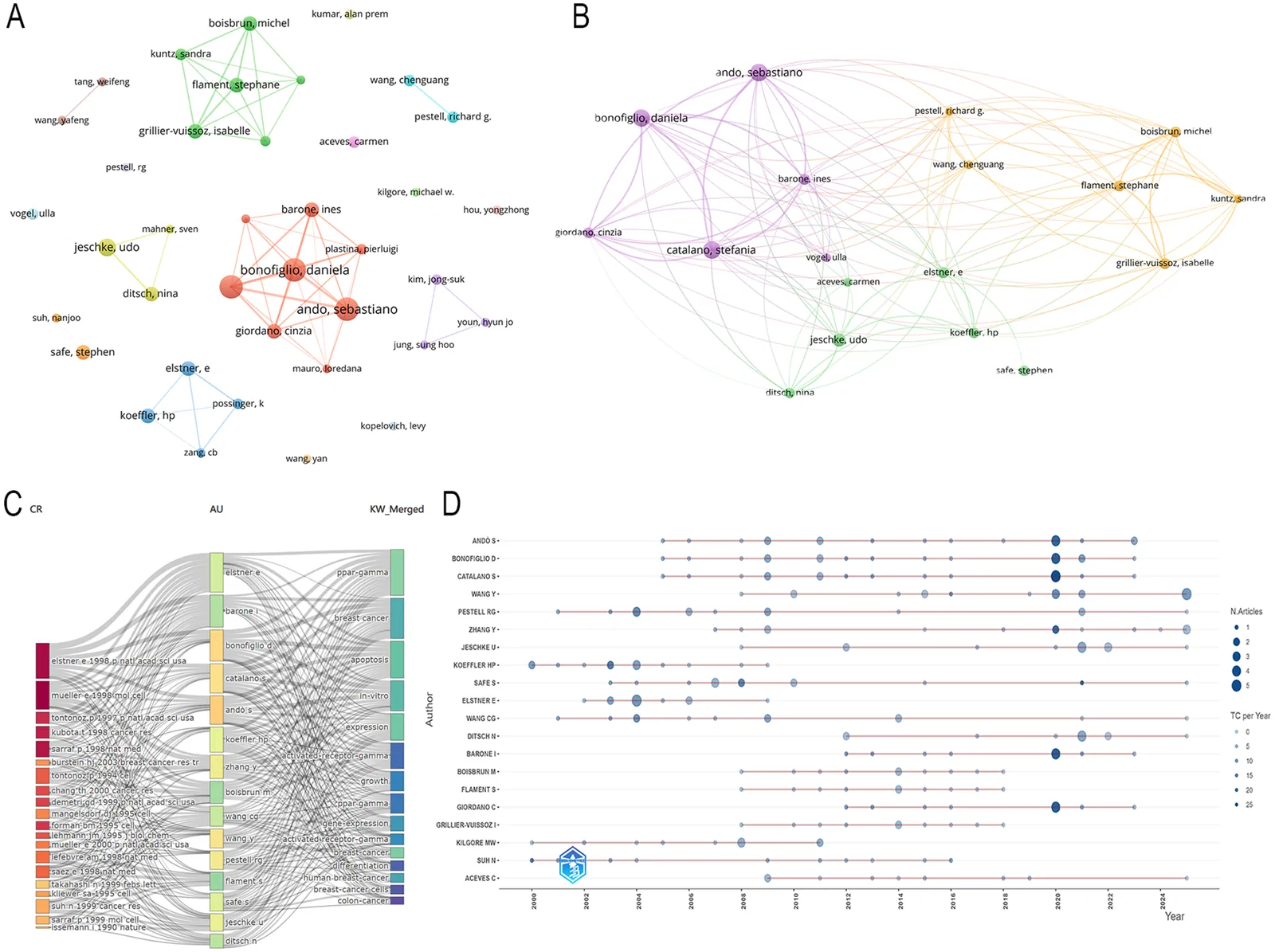
The author’s cooperation network, three-field plot, and production over time. **(A)** The author’s cooperation network. **(B)** The cited author’s cooperation network. **(C)** The three-field plot of CR, AU, and KM_Merged. **(D)** Authors’ Production over Time. The size of the node is directly proportional to the number of published articles. The connection lines represent the existence of cooperative relationships among different authors.

The author collaboration network constructed using VOSviewer revealed that the top four authors with the highest academic contributions were Catalano, Stefania (16 publications), Bonofiglio, Daniela (16 publications), Ando, Sebastiano (16 publications), and Jeschke, Udo (12 publications). A strong intra-group connection exists within the co-author group, indicating that certain authors frequently collaborate and serve as central connectors within the network. This suggests that they play a pivotal role in advancing the research in this subject area. Nonetheless, the overall structure remains fragmented with limited connections among different groups, reflecting a lack of collaboration across various author communities (**Figure 6A**). An analysis of the cited authors indicated that the five authors with the greatest relevance were Catalano, Stefania, Bonofiglio, Daniela, Ando, Sebastiano, Barone, Ines, Giordano, and Cinzia (**Figure 6B**). The three-field plot illustrates the relationships among cited references (CR), authors (AU), and keywords (KM_Merged), effectively visualizing the flow, transformation, and association of core cited literature with key contributors and keywords (**Figure 6C**). The authors’ production over time curve depicts the annual publication trends of the main contributors in this field. Most scholars have been actively engaged in research over an extended period of time, resulting in the production of significant literature (**Figure 6D**).

**Figure 7:**
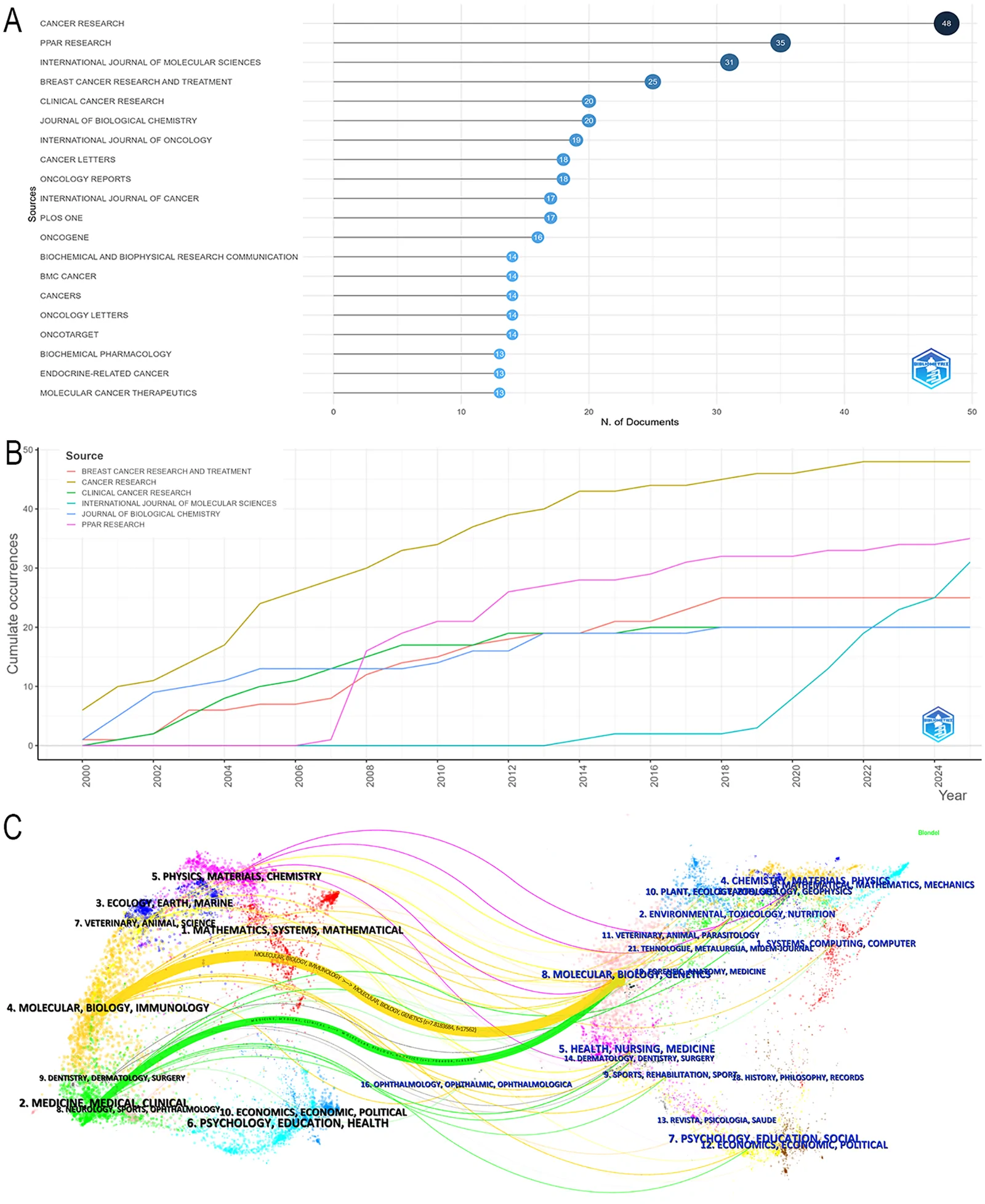
The map of journal production, citation, and knowledge flow. **(A)** Most Relevant Sources. **(B)** Sources’ Production over Time. **(C)** Dual-Map Overlays. Citing Map on the left and Cited Map on the right. Different colors represent the corresponding subject area, and the direction of the connecting line (from right to left) indicates the knowledge flow path; the thickness of the connecting line is directly proportional to the citation strength.

In total, 500 journals have published research in this field. The four journals with the highest number of published articles were CANCER RESEARCH (48 publications), PPAR RESEARCH (35 publications), INTERNATIONAL JOURNAL OF MOLECULAR SCIENCES (31 publications), and BREAST CANCER RESEARCH AND TREATMENT (25 publications). A series of studies published in these journals have established a crucial foundation for a deeper understanding of the role of PPARγ in breast cancer, and have influenced the research trajectory in this area (**Figure 7A**). The trend in the number of journal publications over time indicates that CANCER RESEARCH has consistently maintained a relatively high volume of publications, accompanied by an increasing rate. PPAR RESEARCH has experienced a notable rise in its publication count since 2007, sustaining a relatively high output thereafter. This trend may be closely linked to the 2006 publication and the primary focus on the biological functions of the PPAR family. Since 2019, the INTERNATIONAL JOURNAL OF MOLECULAR SCIENCES has significantly increased its output in this field, demonstrating a continuous upward trend (**Figure 7B**). Dual-Map Overlays are sophisticated visualization tools within CiteSpace that are primarily employed for macro-level analyses of the cross-disciplinary flow and evolutionary trajectory of scientific knowledge. The left side of the view displays the Citing Map, which illustrates the distribution of journals or disciplines in which papers are published. Conversely, the right side presents the Cited Map, depicting the distribution of journals or disciplines associated with cited references. The knowledge base predominantly flows from the MOLECULAR, BIOLOGY, GENETICS to advanced fields such as MOLECULAR, BIOLOGY, IMMUNOLOGY and MEDICINE, MEDICAL, CLINICAL (**Figure 7C**).

**Table 1:** The top ten articles cited the most about PPARγ in breast cancer research.

Rank and strength	Author and journal	Main point
1 (25.86)	Mueller E (26) (MOLECULAR CELL)	The expression level of PPARγ is markedly increased in both primary and metastatic human breast cancer, leading to substantial lipid accumulation and promoting the terminal differentiation of malignant breast epithelial cells. Inhibition of the negative regulatory factor MAP kinase associated with PPARγ can enhance the sensitivity of non-responsive cells to thiazolidinedione (TZD) ligands.
2 (25.86)	Elstner E (27) (PROCEEDINGS OF THE NATIONAL ACADEMY OF SCIENCES OF THE UNITED STATES OF AMERICA)	Combined therapy of Troglitazone and All-trans-retinoic acid induces apoptosis of breast cancer cells through significant down-regulation of Bcl-2.
3 (23.36)	Sung H (28) (CA-A CANCER JOURNAL FOR CLINICIANS)	The global cancer burden has been revised and detailed using the GLOBOCAN 2020 estimates of cancer incidence and mortality published by the International Agency for Research on Cancer. Breast cancer in women has emerged as the most prevalent cancer, with approximately 2.3 million new cases, representing 11.7% of the total. Its mortality rate ranks fifth globally, at 6.9%.
4 (22.63)	Sarraf P (29) (NATURE MEDICINE)	PPARγ is prominently expressed in both well-differentiated and poorly differentiated adenocarcinomas, as well as in normal colonic mucosa and human colon cancer cell lines. PPARγ plays a crucial role in regulating the growth and differentiation of colon cancer cells. Treatment with troglitazone markedly inhibited the growth rate of transplantable tumors derived from human colon cancer cells in murine models.
5 (20.59)	Tontonoz P (30) (PROCEEDINGS OF THE NATIONAL ACADEMY OF SCIENCES OF THE UNITED STATES OF AMERICA)	PPARγ is prominently expressed in the principal histological subtypes of human liposarcoma. Pioglitazone promotes the terminal differentiation of primary human liposarcoma cells. It has been proposed that PPARγ ligands, including thiazolidinediones and retinoid X receptor (RXR) -specific vitamin A, may serve as effective therapeutic agents for the treatment of liposarcoma.
6 (19.50)	Burstein HJ (31) (BREAST CANCER RESEARCH AND TREATMENT)	To evaluate the therapeutic effects of troglitazone in patients with refractory metastatic breast cancer. For patients with advanced breast cancer who exhibit resistance to at least one chemotherapy regimen (for ER-negative tumors) or two hormone regimens (for ER-positive tumors), troglitazone is administered at a dosage of 800 mg orally once daily until disease progression occurs. While clinical trials involving various patient populations may indicate minor effects on tumor differentiation, the activation of PPARγ by troglitazone lacks significant clinical relevance in the treatment of patients with refractory metastatic breast cancer.
7 (18.95)	Kubota T (32) (CANCER RESEARCH)	Human prostate cancer cells exhibit elevated levels of PPARγ, whereas normal prostate tissue shows minimal expression. PPARγ ligands, including troglitazone, have been shown to inhibit the growth and proliferation of prostate cancer cell lines without impacting the cell cycle or differentiation, thereby reducing the degree of malignancy.
8 (17.27)	Saez E (33) (GENES & DEVELOPMENT)	Transgenic mice that express constitutively active PPARγ in their mammary glands, when crossed with transgenic strains predisposed to breast cancer, develop tumors with significantly accelerated progression and increased malignancy. These dual-gene tumors may promote elevated secretion and differentiation levels through the activation of Wnt signaling pathways.
9 (16.79)	Grommes C (34) (LANCET ONCOLOGY)	PPAR is a ligand-activated transcription factor that comprises three distinct subtypes: PPARα, PPARγ, and PPARβ/δ. Each subtype heterodimerizes with the 9-cis-retinoic acid receptor RXR and plays a crucial role in regulating various metabolic pathways, including lipid biosynthesis and glucose metabolism. Additionally, they induce anti-tumor and anti-inflammatory effects in diverse mammalian cells.
10 (16.07)	Yee LD (35) (CLINICAL CANCER RESEARCH)	Short-term treatment with rosiglitazone (8 mg/d) did not significantly affect the proliferation of breast cancer tumor cells, as indicated by the Ki-67 index.

**Figure 8:**
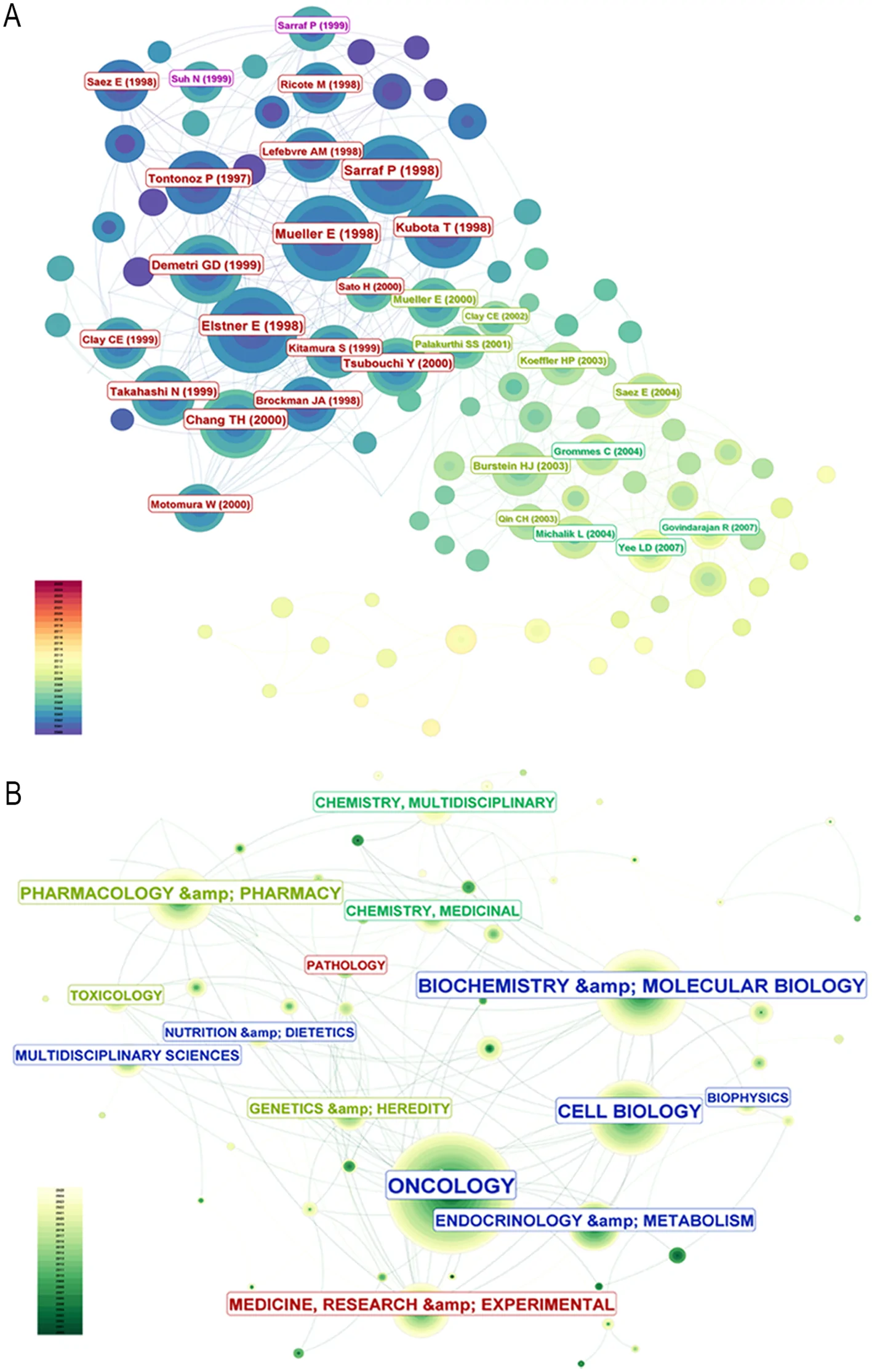
The visual knowledge map of reference and category analysis. **(A)** The visual knowledge map of reference analysis. **(B)** The visual knowledge map of category analysis. The larger the node, the greater the number of published articles. The connection lines between nodes indicate the existence of associations.

**Figure 9:**
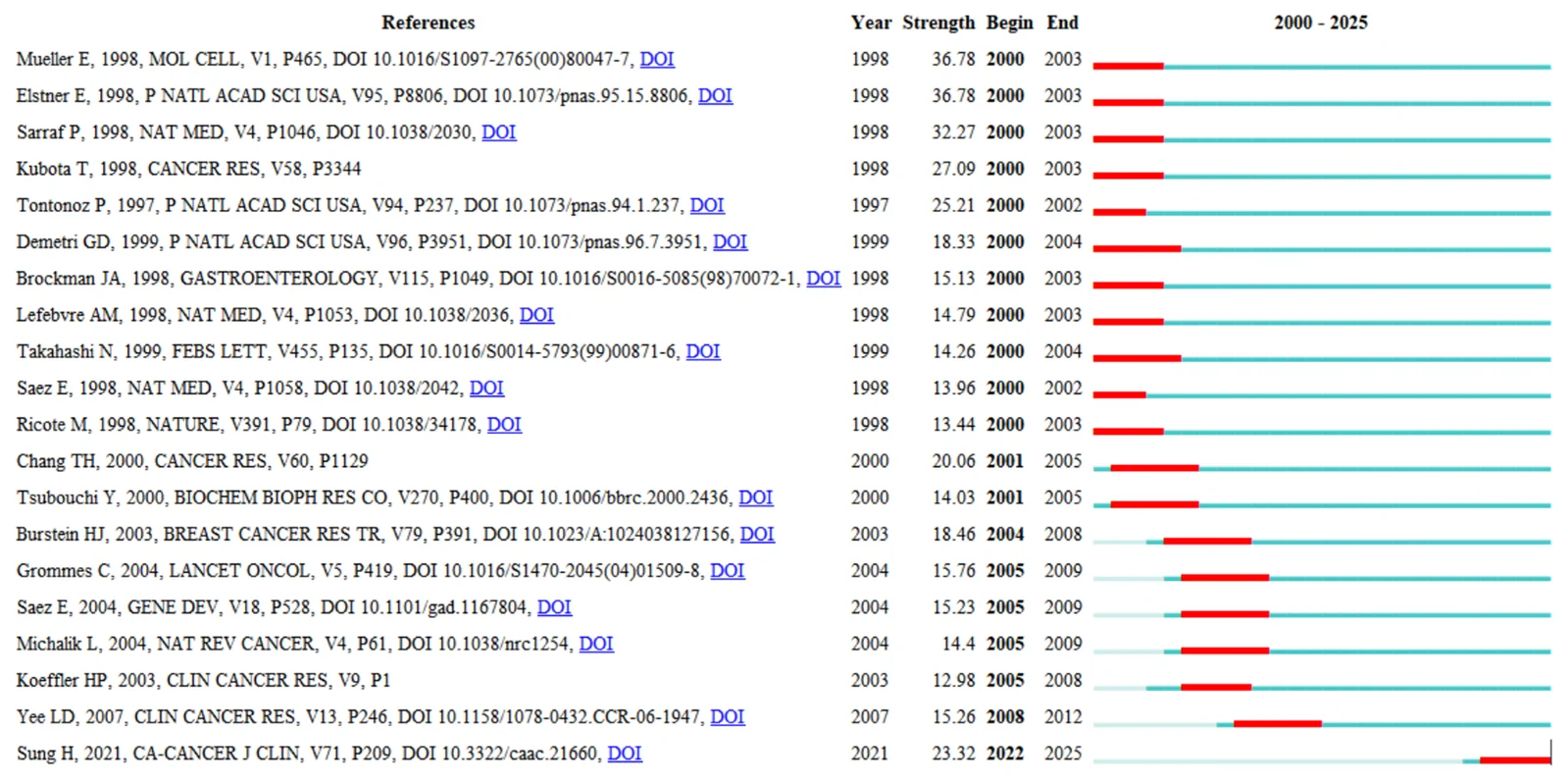
Top 20 references with the strongest citation bursts. The vertical axis lists the keywords that occur frequently. The horizontal axis (2000-2025) represents the time axis. The light blue bars in the bar chart represent the basic presence of the keyword throughout the research period. The solid red bar represents the peak period of this keyword in a specific year range.

### 3.4 References and category analysis

Citing references serves as the foundation and vehicle for a discipline's knowledge system. Systematic literature citations facilitate the establishment of a relatively stable disciplinary framework and a general consensus. References concerning PPARγ expression in breast cancer were visualized (**Figure 8A**). Additionally, an analysis was conducted on the top 20 references that exhibited the strongest citation bursts (**Figure 9**). A systematic review of the ten most highly cited studies (**Table 1**) indicated that PPARγ comprehensively regulates metabolic pathways and biological behaviors, including the growth, proliferation, and differentiation of breast cancer cells. The application of PPARγ agonists such as troglitazone and rosiglitazone has been shown to promote apoptosis in breast cancer cells. Furthermore, PPARγ has complex regulatory effects in liposarcoma, prostate cancer, and colon cancer. Category analysis primarily elucidates the proximity and evolution of interdisciplinarity by examining disciplinary categories associated with the literature. The disciplinary visualization of PPARγ within breast cancer research (**Figure 8B**) demonstrates that key disciplinary nodes, including ONCOLOGY, BIOCHEMISTRY and MOLECULAR BIOLOGY, and CELL BIOLOGY, occupy significant positions and are intricately linked, thereby forming a cooperative network. This underscores the highly interdisciplinary nature of the field.

### 3.5 Keyword analysis

**Figure 10:**
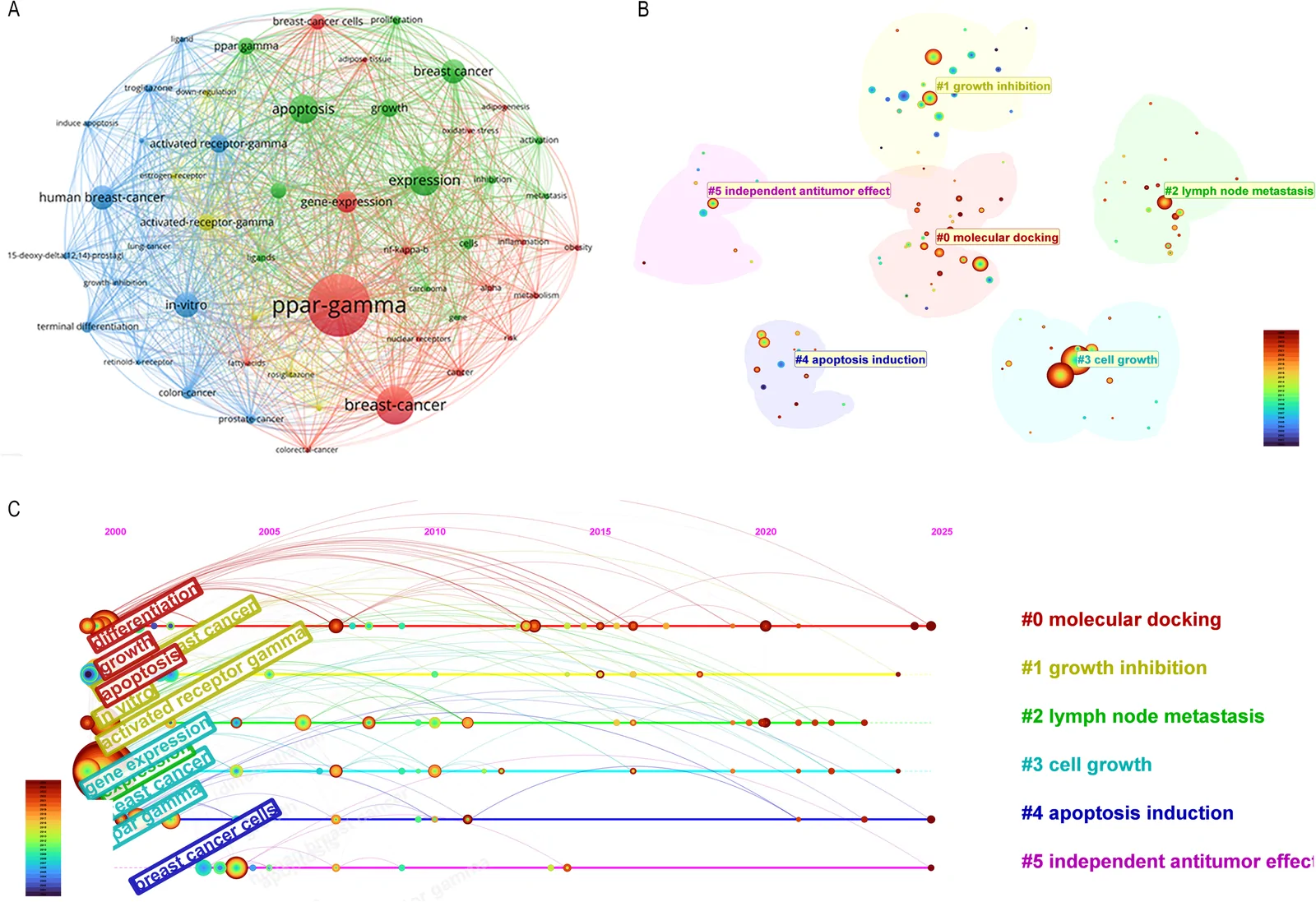
The visualization knowledge map of keywords. **(A)** Network visualization of high-frequency keywords. **(B)** Keyword cluster analysis. **(C)** Keyword clusters timeline analysis. The size of a node is positively correlated with the number of published articles, and the connection lines between nodes indicate the existence of a connection.

**Figure 11:**
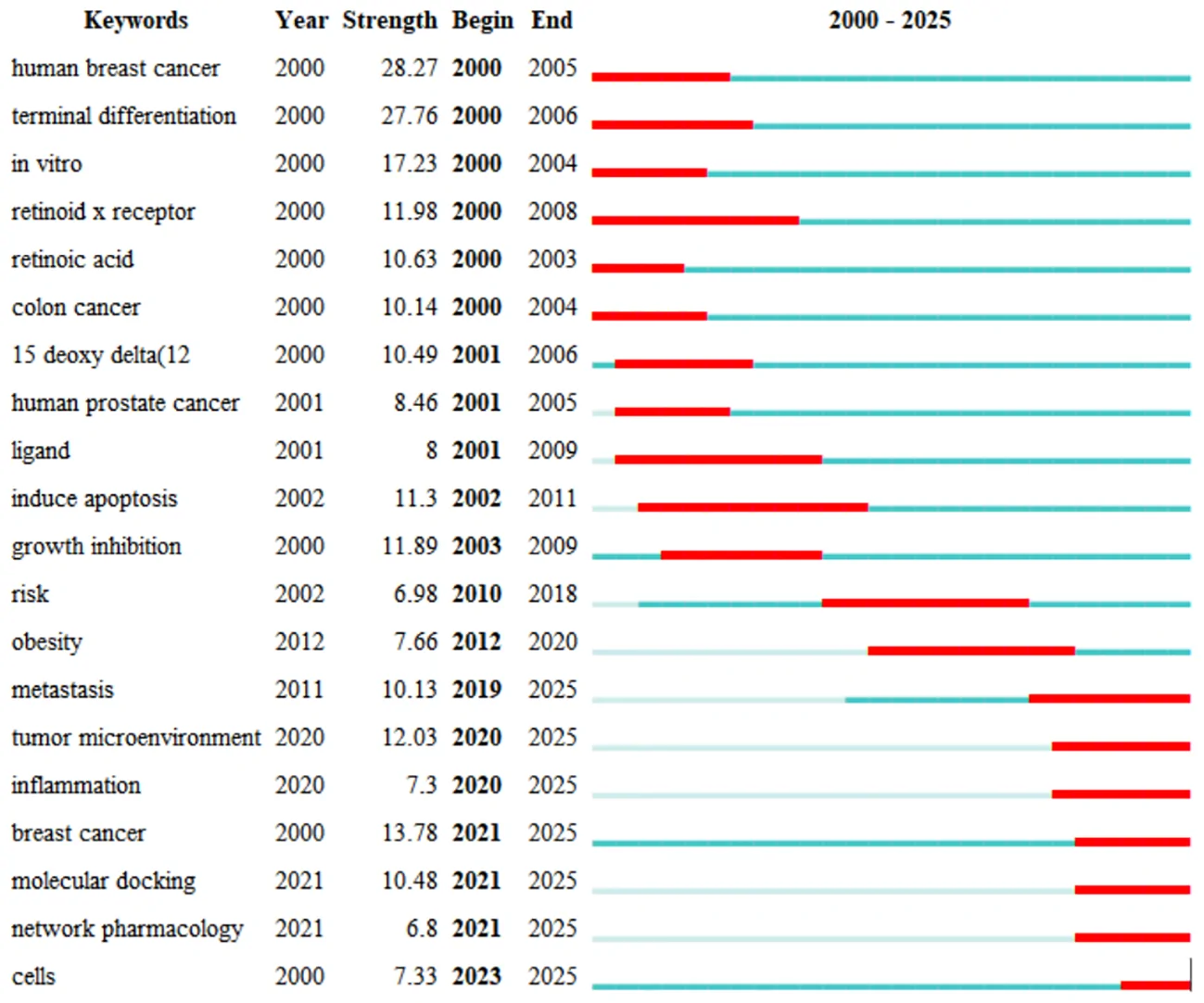
Top 20 keywords with the strongest citation bursts. The vertical axis lists the keywords that occur frequently. The horizontal axis (2000-2025) represents the time axis. The light blue bars in the bar chart represent the basic presence of the keyword throughout the research period. The solid red bar represents the peak period of this keyword in a specific year range.

Keyword co-occurrence analysis serves as an efficient and intuitive method for elucidating knowledge structure, research hotspots, and evolutionary context within a specific research field. The keyword co-occurrence analysis (**Figure 10A**) pertaining to PPARγ in breast cancer indicated that the most frequently occurring keywords were PPARγ, breast cancer, apoptosis, and expression. This finding suggests that most literature concentrates on these terms for research purposes. Additionally, keyword cluster analysis (**Figure 10B**) identified six keyword clusters, which include molecular docking, growth inhibition, lymph node metastasis, cell growth, apoptosis, induction, and independent antitumor effect. The timeline analysis (**Figure 10C**) illustrated the temporal evolution of keyword clustering, thereby highlighting the developmental trajectory of the research topic and its progression from basic medicine to precise clinical treatment. An emergent analysis of the keywords was performed, which resulted in the extraction of the top 20 keywords with the highest citation counts (**Figure 11**). This analysis indicates a growing incorporation of regulatory mechanisms within the research field, reflecting an evolutionary process. The focus shifted from terminal differentiation, induction of apoptosis, and inhibition of oxidative stress, inflammation, and proliferation. Concurrently, novel research methodologies, such as molecular docking and network pharmacology have enhanced traditional studies.

### 3.6 Thematic evolution and changing trend

**Figure 12:**
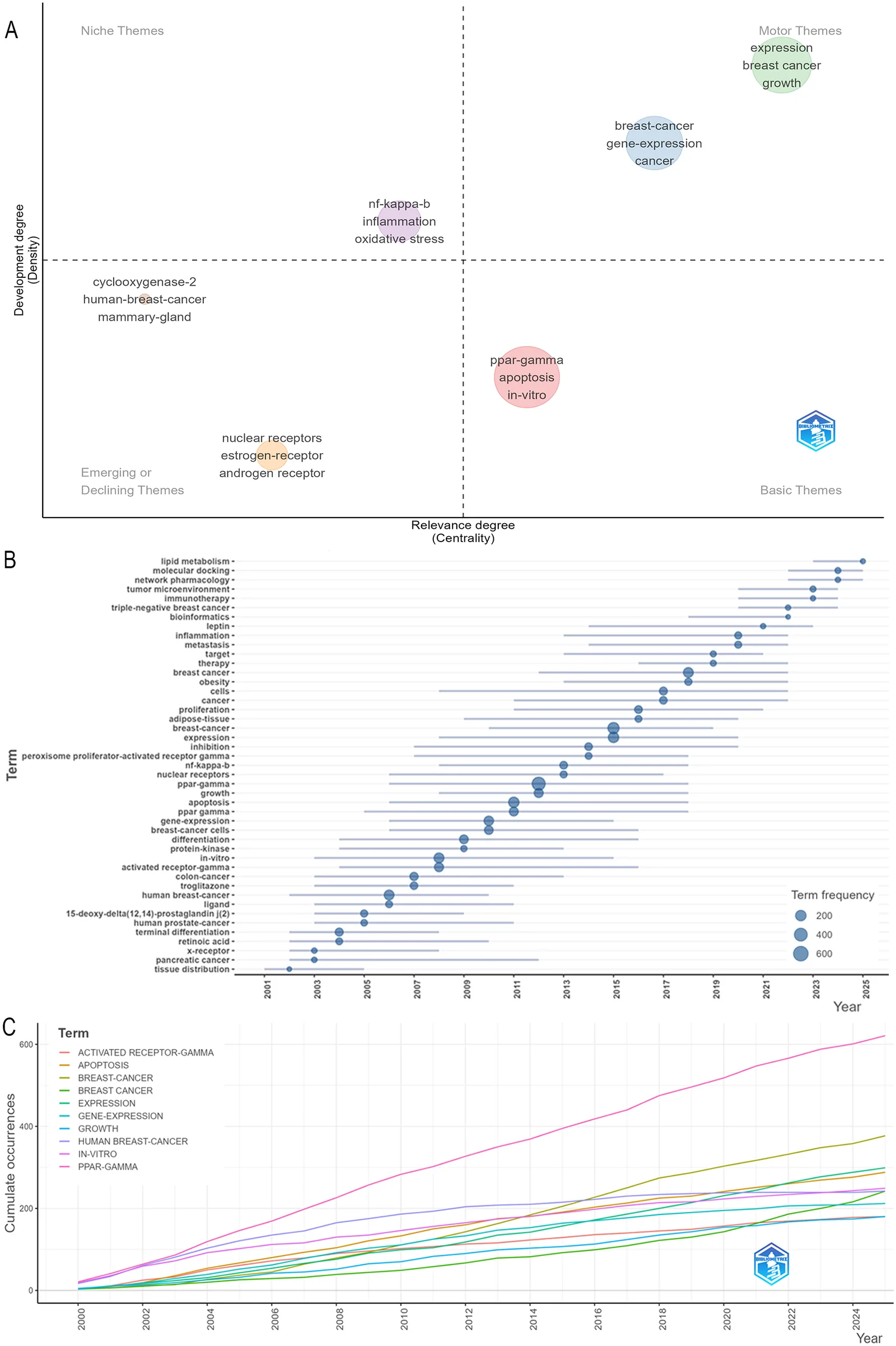
The visualized knowledge map of thematic evolution and changing trends. **(A)** Thematic map. **(B)** Trend topics. **(C)** Words’ frequency over time.

Thematic maps position research topics along coordinate axes based on their centrality and density, dividing them into four quadrants (**Figure 12A**). PPARγ, apoptosis, and *in vitro* studies have served as the foundational themes. Core themes, such as breast cancer, gene expression, cancer, and growth drive the development and evolution of research. Quadrants representing emerging or declining themes included nuclear receptors, estrogen receptors, androgen receptors, and cyclooxygenase-2. This may be due to a deeper understanding of the basic physiological functions of PPARγ. Additionally, recently discovered cyclooxygenase-2 has made significant strides in regulating the tumor microenvironment in breast cancer through its synergistic effects with PPARγ. Trend topics illustrate the shifting trends of research topics over the years (**Figure 12B**), evolving from basic biological behaviors, such as regulating terminal differentiation, to complex mechanisms involving lipid metabolism, inflammation, and the tumor microenvironment. This regulatory model exemplifies the core characteristics of the coordinated integration of multiple signaling pathways and the precise targeting of effects, which together establish the molecular basis for high efficiency and specificity. The term “frequency over time” visualizes the occurrence of subject words over time (**Figure 12C**).

### 3.7 Sensitivity analysis

assess the robustness of the analysis results, the parameters were perturbed by incrementally increasing the value of the g index k to perform a sensitivity analysis. For citation emergent references, the repetition of the top 20 emergent references across varying k values was examined. A total of 14 references were identified as repeated, yielding a repetition rate of 70.00%. For keyword emergence, the repetition of the top 20 keywords was analyzed using the same methodology. A total of 15 keywords were found to be repeated, resulting in a repetition rate of 75.00%. These findings suggest that the primary emergent objects and the core clustering structure exhibit high consistency across different parameter settings, thereby confirming that the analysis results of this study are not sensitive to parameter selection and demonstrate substantial robustness.

**Figure 13:**
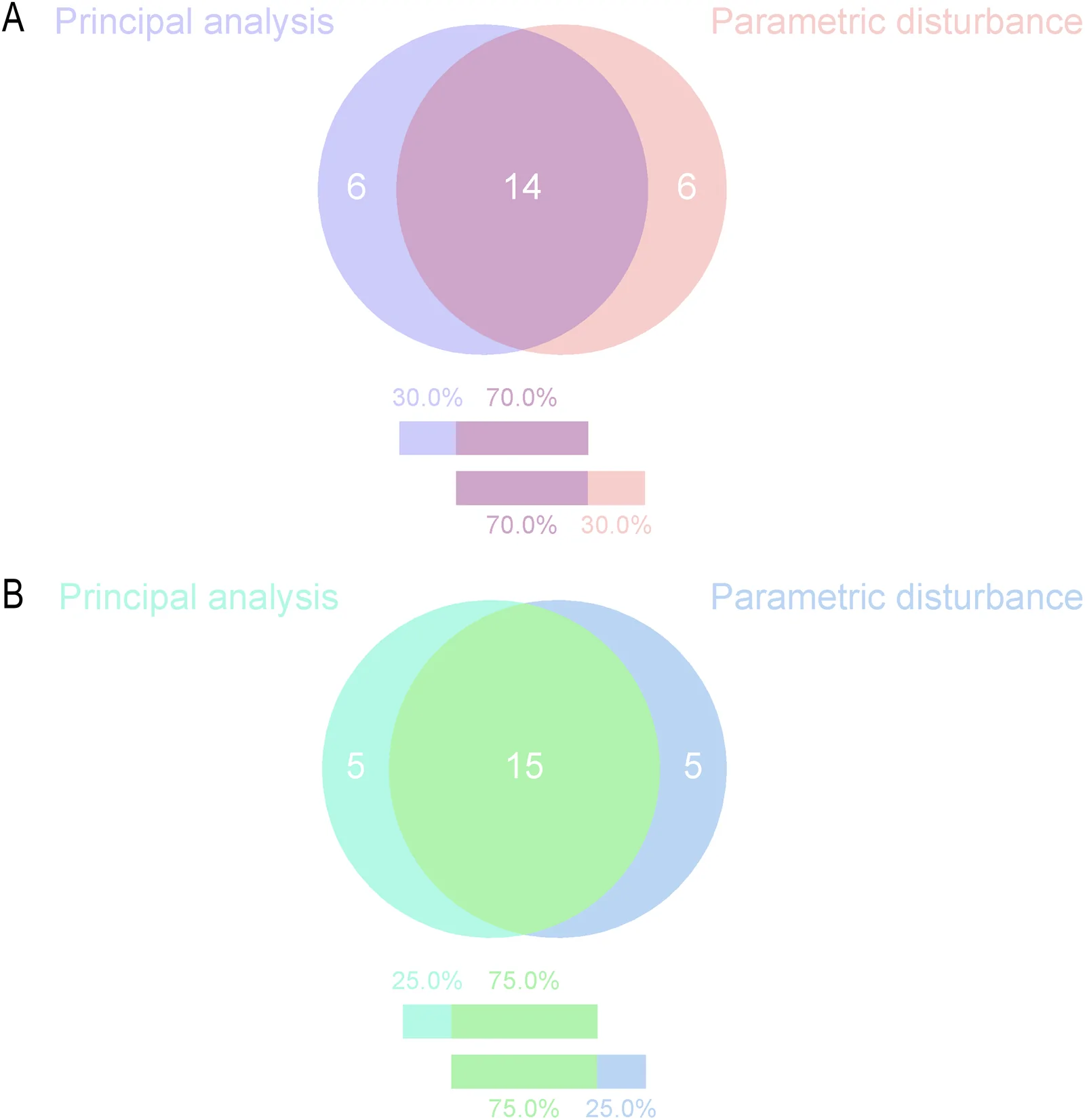
Venn diagram of sensitivity analysis. **(A)** Venn diagram of references. **(B)** Venn diagram of keywords. The data sets of principal analysis and parameter disturbance intersect, the middle represents the intersection, and the bar chart shows the proportion.

### 3.8 RCTs analysis

**Figure 14:**
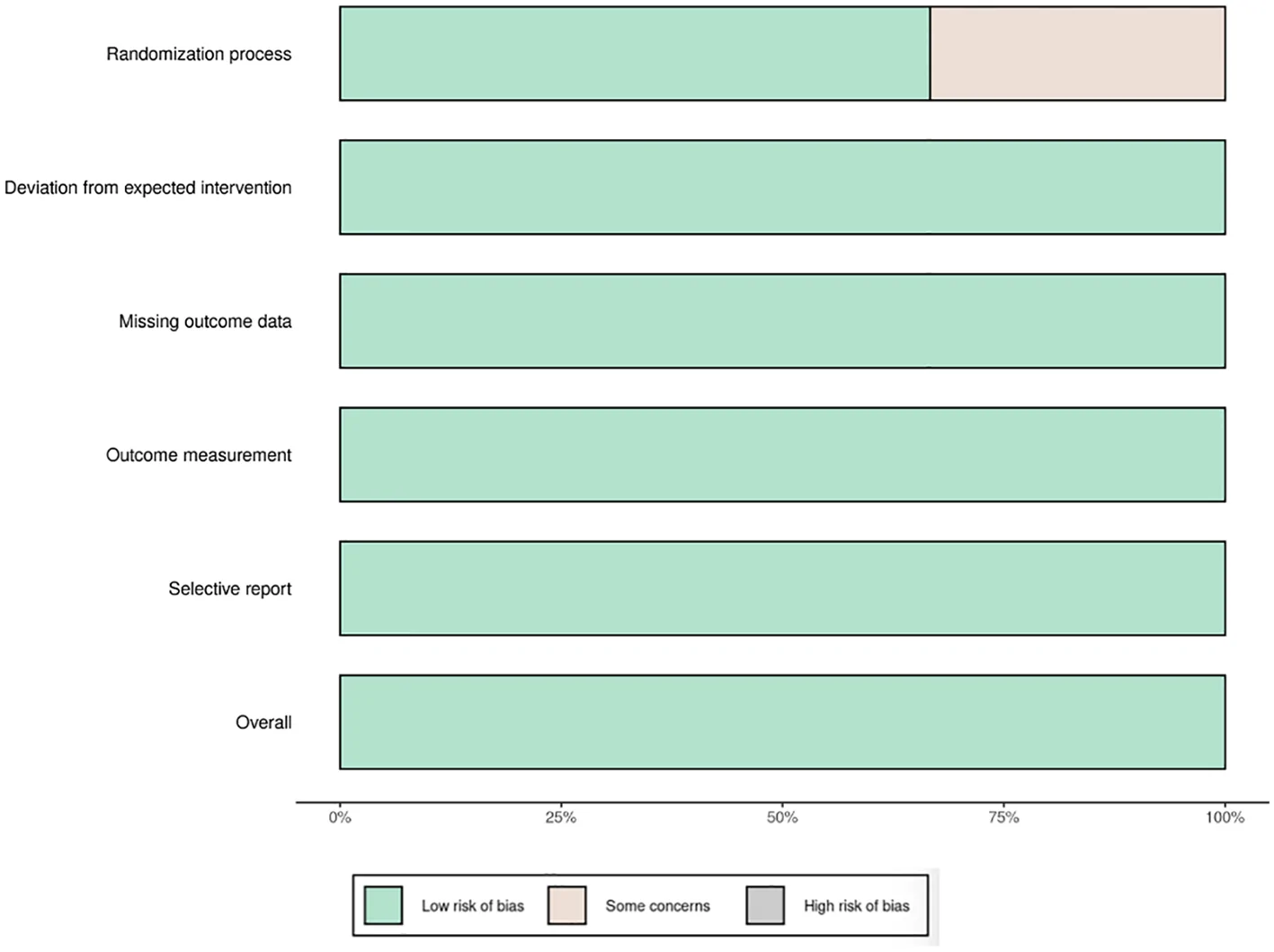
Based on Cochrane RoB 2 tool, the risk map of publication bias assessment for three RCTs. The horizontal axis represents the percentage of different literature sources, and the vertical axis represents different evaluation items. Green represents low risk, red represents worrying risk, and gray represents high risk.

Four RCTs were identified from the PubMed database. Following the exclusion of one study that was unrelated to this field, three RCTs were included in the analysis (**Table 2**). The publication bias of these three studies is evaluated based on Cochrane RoB 2 tool, and the results indicate that these three studies have a low risk of publication bias (**Figure 14**). These studies have employed rigorous scientific methodologies, varied research designs, sample sizes, and intervention strategies. Key findings indicated that individuals with the PPARG Pro 12 Ala wild-type genotype exhibited a significantly elevated risk of breast cancer. Furthermore, PPARγ serves as a critical upstream regulatory gene in lipid metabolism associated with breast cancer, and is strongly correlated with prognosis. This may represent a potential therapeutic target for HR-positive and HER2 negative breast cancer. Additionally, high expression levels of PPARγ appear to inhibit the malignant transformation of benign breast lesions.

**Table 2:** Summary of RCTs of PPARγ in the field of breast cancer research.

References	Trial registration	Study design	Participants	Intervention	Primary outcomes
Kopp et al., 2016 (36)	NCT02463383	A randomised, double-blinded, placebo controlled 2x24 h crossover study.	From December 1993 to May 1997, there were 29,875 women aged 50-64 who were born in Denmark and lived in Copenhagen or Aalborg, and had no history of cancer at the time of invitation.	48 hours before each intervention, participants were asked to refrain from alcohol consumption and any form of painkillers. The participants showed up fasting and were served lunch at the University’s dining facility 1½ hours before drinking. 30 min prior to drinking, participants had their Ibuprofen or placebo tablet administered.	Regardless of CYP19A1 genotype, PPARG Pro 12 Ala wild-type carriers have a significantly increased risk of breast cancer.
Liang et al., 2018 (37)	NCT00629616	The CARMINA 02 phase 2, multicenter, open-label, non-comparative, randomized trial.	116 postmenopausal women with operable breast cancer	59 in the anastrozole arm (1 mg daily) and 57 in the fulvestrant arm (500 mg with a loading dose on day 1, 15, and 29 for the first month and then every 4 weeks).	Lipid metabolism is the most important molecular function related to prognosis, and PPARγ is the most important upstream regulatory gene, which can be used as a potential therapeutic target for HR-positive and HER2-negative breast cancer.
Pirouzpanah et al., 2019 (38)	IRCT2012110511335N2	Randomized, double-blind, placebo-controlled clinical trial.	85 pre-menopausal women afflicted with BBD diagnosed by ultrasonography imaging aged between 19 and 52 years old. They enrolled from May 2013 to October 2014 in Tabriz, Eastern Azerbaijan, Iran.	They were assigned randomly into either the BV juice group (n = 44, BV juice: 480 ml/day) or the placebo group (n = 41, BV placebo juice: 480 ml/day) for 8-week intervention.	PPARγ was significantly higher in the placebo group than in the intervention group. Highlighting the possible inhibitory effects of BV juice on malignant transformation for benign breast disease.

## 4 Discussion

### 4.1 The academic pattern of PPARγ in the field of breast cancer research

The bibliometric analysis conducted in this study reveals that the United States, China, Japan, and Italy are the four countries with the highest publication output and citation counts, indicating that their academic research contributions are well recognized within the scholarly community. The cooperative world map illustrates the extensive academic connections among countries globally. The United States, positioned as the hub of academic exchange and collaboration, has spearheaded the advancement of various disciplines. Notably, Harvard University, the University of Texas MD Anderson Cancer Center, and the National Cancer Institute, which are the three institutions with the highest number of published papers, are all located in the United States, underscoring the nation’s robust scientific research capabilities in this domain. In 1991, the National Institutes of Health in the United States published a consensus conference on the treatment of breast cancer, which had a significant academic impact. As a crucial component of the evolution of academic concepts, it has also transformed surgical approaches (39). However, numerous regions worldwide remain underexplored, reflecting a deficiency in relevant research foundations and international collaboration. It is imperative to enhance the intensity of international cooperation to foster a greater understanding and research on PPARγ in breast cancer.

The three authors with the highest academic contributions—Catalano, Stefania; Bonofiglio, Daniela; and Ando, Sebastiano—are affiliated with the University of Calabria in Italy. These authors also exhibited the highest mediating centrality among the cited scholars. A robust intra-group connection exists among them, which has significantly influenced the advancement of the research theme. Nevertheless, the overall structure remains fragmented, indicating a continued need for enhanced collaboration among the authors.

Analysis of the top ten references with the highest emergence intensity indicated that PPARγ played a crucial role in regulating metabolic pathways and biological behaviors, including growth, proliferation, and differentiation. Targeting the PPARγ pathway may enhance apoptosis in breast cancer cells. Furthermore, its involvement has been validated in liposarcoma, prostate cancer, and colon cancer.

The most frequently occurring keywords were PPARγ, breast cancer, apoptosis, and expression. The extracted keyword clusters encompassed molecular docking、 growth inhibition、lymph node metastasis、cell growth、apoptosis、induction、independent antitumor effect. This highlights the importance of the intricate regulation of gene expression and various pathophysiological processes within this research domain. Furthermore, it has become increasingly evident that PPARγ may play a critical role in the regulation of breast cancer through newly identified mechanisms and pathways, thereby establishing a complex network involving cancer, metabolism, and immunity. Advances in technologies and methodologies have propelled further progress in this field, enabling the development of precise and targeted clinical interventions.

### 4.2 The molecular mechanism of PPARγ regulating lipid metabolism in breast cancer

PPARγ is a crucial member of the nuclear receptor superfamily. It is involved not only in the regulation of glycolipid metabolism but also in metabolic reprogramming associated with breast cancer. PPARγ activity is modulated by both natural and synthetic ligands (40). Upon forming a heterodimer with the retinol X receptor, the complex is recruited to the PPAR response element (PPRE) located in the promoter region of the target gene, thereby modulating its transcription (41). In breast cancer cells, highly expressed PPARγ translocates to the nucleus and binds to Nur77, facilitating the recruitment of ubiquitin ligases that promote ubiquitination and subsequent degradation of Nur77. As a tumor suppressor, Nur77 inhibits transcription by recruiting the SWI/SNF complex to the promoter regions of CD36 and FABP4, thereby obstructing the uptake of exogenous fatty acids by cancer cells (42). Targeting and obstructing the interaction between Nur77 and PPARγ, while simultaneously restoring the expression and function of Nur77 can effectively inhibit lipid uptake, cellular proliferation, and bone metastasis in breast cancer cells (43). PPARγ activation can upregulate the expression of fatty acid synthase (FASN) and acetyl-CoA carboxylase (ACC), thereby regulating *de novo* synthesis of fatty acids (44).

PPARγ agonists have demonstrated potential in preclinical studies to inhibit the proliferation of breast cancer cells, induce apoptosis, and suppress metastasis (45). Yee reported that the therapeutic effect in clinical studies was unsatisfactory (35). This may be attributed to the subtype-specific and context-dependent nature of PPARγ, which can promote tumor growth in certain environments. Consequently, future treatment approaches should aim to achieve greater precision by examining the specific functional network of PPARγ in various breast cancer subtypes and microenvironments. The development of highly selective targeted therapies is crucial, ultimately translating this “metabolic checkpoint” into an effective clinical treatment strategy.

### 4.3 The role of PPARγ in regulating cancer progression in breast cancer

PPARγ demonstrates a notable background-dependent dual effect in breast cancer, which is influenced by subtype, disease stage, and TME. In estrogen receptor-positive (ER-positive) breast cancer, PPARγ generally functions as a tumor suppressor, inhibiting tumor growth by disrupting the estrogen receptor signaling pathway, inducing cell cycle arrest, and promoting cellular differentiation (46). However, in TNBC, PPARγ may exert tumor-promoting effects by promoting lipid metabolism reprogramming and characteristics of tumor stem cells (47). In early-stage breast cancer, higher PPARγ expression is usually associated with better prognosis, and high PPARγ activity in advanced breast cancer may be associated with enhanced metastatic ability (48, 49). This contradictory effect arises in part from the differential expression and function of PPARγ across various cell types. Research involving a specific PPARγ knockout in breast epithelial cells indicated that PPARγ expression in these epithelial cells may inhibit the early stages of tumor development, whereas its activation in tumor cells may facilitate progression (50).

PPARγ modulates breast cancer progression through various pathophysiological mechanisms. Upon ligand activation, PPARγ inhibits cell growth, reduces tumor invasiveness, and diminishes epithelial to mesenchymal transition (EMT) in breast tissues (51). In addition to its role in regulating the metabolism of breast cancer cells, PPARγ modulates immune cells, stromal cells, and other components of the TME. This regulation subsequently influences the growth, invasion, and metastasis of breast cancer (47, 52). Studies have demonstrated that the inhibition of PPARγ activation and β-catenin-mediated M2 macrophage polarization can regulate the downstream NF-κB signaling pathway. This regulation promotes the polarization of the M1 phenotype, thereby inhibiting breast cancer growth and macrophage infiltration (53). Stromal Derived Factor-1α (SDF-1α) and its receptor CXCR4 are crucial in mediating the invasion and metastasis of breast cancer cells. PPARγ recruits the silencing mediator of retinoid and thyroid hormone receptor (SMRT) co-suppressor to the PPRE of the CXCR4 promoter in breast cancer cells, thereby down-regulating its transcriptional activity. CAFs secrete elevated levels of SDF-1α. Following activation of PPARγ by rosiglitazone, the CXCR4 signaling pathway induced by CAFs was diminished, resulting in reduced motility and invasiveness, as well as morphological changes. These findings suggest the potential application of PPARγ in inhibiting breast cancer stromal cells (54). In addition, some special protein modifications and regulations also mediate the anti-breast cancer function of PPARγ, such as acetylation (55).

### 4.4 The potential value of PPARγ in the surgical treatment of breast cancer

The prognostic value of PPARγ expression in patients with breast cancer is significantly influenced by the molecular subtype of the disease. PPARγ is a potential biomarker for predicting the 5-year recurrence rate and is associated with the cancer aggressiveness index (Ki-67) and histological grade. Furthermore, it exhibits a positive correlation with ERβ and serves as an independent prognostic indicator in patients diagnosed with ductal carcinoma (56, 57). These findings further substantiate the potential utility of PPARγ as a biomarker for prognostic assessment of breast cancer. By modulating lipid metabolism pathways in breast cancer cells and the TME, PPARγ activation can impede tumor growth, proliferation, metastasis, and invasion at the metabolic level, which is crucial for establishing favorable conditions for surgery (53, 58).

Numerous preclinical studies have demonstrated that PPARγ activation can inhibit tumor growth, induce cell cycle arrest, and promote apoptosis. This finding holds considerable significance for clinically reducing the tumor volume, lowering the clinical stage, and ultimately enhancing the success rate of radical surgical resection or breast-conserving surgery. These results suggest that PPARγ targeting represents a potential neoadjuvant treatment strategy. However, the clinical efficacy of PPARγ agonists in breast cancer remains unclear. Burstein reported that troglitazone lacks significant clinical value in the treatment of patients with refractory metastatic breast cancer (31). When traditional TZD drugs exert therapeutic effects, they may simultaneously activate beneficial transcription programs associated with efficacy in the PPARγ signaling pathway, along with non-target transcription programs linked to adverse reactions, such as weight gain and edema (59, 60). Developing a new generation of selective PPARγ modulators that can effectively distinguish target activity from traditional side effects at the transcriptional level represents a crucial direction for future advancements in this field. The mode of interaction of INT131 with PPARγ receptor markedly differs from that of conventional TZD drugs. This distinct interaction ultimately leads to the recruitment of co-regulatory factors and alters the gene transcription profiles. In diabetes-related clinical trials, INT131 has shown significant advantages in controlling blood sugar and lipid levels, while also mitigating common side effects associated with TZDs, such as weight gain and edema (61). Given its enhanced pharmacological properties, the potential role of INT131 in breast cancer treatment requires further investigation.

The integration of PPARγ agonists with chemotherapy, targeted therapy, or immune checkpoint inhibitors to enhance therapeutic efficacy and mitigate drug resistance represents a primary avenue of current research. Studies have indicated a significant positive correlation between PPARγ expression and expression of various immune-related genes and immune checkpoint molecules. Further observations suggest that patients with ER+ tumors demonstrate improved therapeutic responses to immune checkpoint blockade therapy (62). PPARγ expression may be associated with drug resistance during treatment. This mechanism likely involves the regulation of Akt phosphorylation and expression of apoptosis-related proteins, including Bcl-2 (63). In ER+ and tamoxifen-resistant breast cancer, cystathionine-γ-lyase (CSE) facilitates tumor growth and invasion by modulating PPARγ sulfhydration, thereby affecting its expression. The CSE inhibitor I194496 has the potential to reverse tamoxifen resistance in breast cancer by targeting the PPARγ/ACSL1/STAT3 signaling pathway (64).In contrast to chemotherapy alone, incorporation of pioglitazone with cisplatin significantly diminished the viability of breast cancer cells and increased cell apoptosis. This finding underscores the potential role of PPARγ activation in metabolic sensitization (45). Doxorubicin is a potent chemotherapeutic agent for breast cancer; however, its clinical use is frequently limited by considerable cardiotoxicity. Concurrent administration of the PPARγ agonist rosiglitazone can significantly decrease the effective therapeutic dose of doxorubicin to one-twentieth of the dose used in isolation, thereby mitigating its adverse cardiac effects (65).

### 4.5 Analysis of limitations

This study’s data collection and analysis process were subject to several limitations, each accompanied by relevant explanations. First, regarding literature sources, the bibliometric analysis conducted using CiteSpace and VOSviewer primarily relied on the WOSCC. This choice was made because these analysis tools exhibit optimal compatibility with the literature format of this database, thereby ensuring both the stability of the analysis process and interpretability of the results (25). To mitigate the potential issue of incomplete literature coverage associated with a single database, this study supplemented its collection of relevant RCTs published in the PubMed database, thereby enhancing the comprehensiveness of clinical evidence. Second, with regard to language selection, this study only included literature published in English. While this approach may have excluded some studies published in other languages, introducing a degree of selection bias, it is anticipated that this will not significantly affect the overall research trend or the primary conclusions, given that English serves as the predominant academic language in this field, and the volume of non-English literature is relatively limited. In terms of quality control, this study did not employ a systematic evaluation process for the included literature, which may have influenced the strength of the evidence presented. Nevertheless, during the literature import and preprocessing phase, the study utilized the Bibliometrix tool was used to automatically retrieve and verify fundamental information from the literature. To some extent, this approach ensured the basic quality and consistency of the overall literature set. The primary objective of this study was to conduct a systematic knowledge graph visualization analysis of the role of PPARγ in breast cancer for the first time, thereby providing a structured context for knowledge evolution in this field. Future research should aim to optimize the literature screening strategy, establish a standardized inclusion and quality evaluation system, and broaden literature sources across multiple languages and databases. Such efforts would facilitate a more comprehensive and systematic understanding of the development trends and research frontiers in this area.

## 5 Conclusion

This study adopted bibliometric methods to systematically track and retrospectively analyze the progress of PPARγ research in the field of breast cancer since 2000, clarifying the research hotspots, development trends, and dynamic evolution of the academic landscape in this field. Research findings indicate that global-related studies are mainly led by countries such as the United States, Italy, and China and that there is a clear imbalance in international cooperation and development. By sorting out the ten literatures with the highest intermediary centrality in the cited networks, the study further identified the core consensus and knowledge basis in this field. Preclinical studies indicate that PPARγ is markedly expressed in breast cancer cells, regulates various biological processes, contributes to the formation of the “cancer-metabolism-immunity” interaction network, and plays a multifaceted role in tumor initiation and progression. Observational studies reveal that PPARγ activation does not exhibit significant clinical relevance in treating patients with refractory metastatic breast cancer. Moreover, this study synthesizes clinical evidence from RCTs, suggesting that wild-type carriers of PPARG Pro12Ala demonstrate increased susceptibility to breast cancer, with PPARγ serving as a crucial upstream gene that regulates lipid metabolism in this context. Its expression correlates with patient prognosis and may represent a potential therapeutic target for HR-positive and HER2-negative breast cancer. These findings effectively bridge basic mechanistic research with clinical insights and provide a substantial foundation for the clinical translation of PPARγ-related biological theories. They hold considerable translational medical value for the development of selectively activated PPARγ drugs and the formulation of precise treatment strategies for breast cancer that are based on lipid metabolism.

## Data Availability

The original contributions presented in the study are included in the article/**Supplementary Material**. Further inquiries can be directed to the corresponding authors.

## Supplementary material

Supplementary Figure 1 Increasing the k-value (1), the top 20 references with the strongest citation bursts analysis.

Supplementary Figure 2 Increasing the k-value (1), the top 20 keywords with the strongest citation bursts analysis.
